# A Sanatorium Christmas in the North

**Published:** 1913-01-04

**Authors:** 


### A SANATORIUM CHRISTMAS IN THE NORTH.

What has proved the most abiding remembrance of Christmas in a large hospital was the sight of an overjoyed child. On his little fair head, fitting it all round like a glove, sat a paper cap from a cracker. The procession of house surgeons playing ragtime had just passed, and the little six-year-old, with that intense delight on his face we only feel in childhood, was waving a small arm in exultation.

This—the sight of children's pleasure—is about the only reward responsible people get for their trouble at Christmas time. Our good friends the idealists will excuse us for maintaining that it *is* a troublesome time. There are entertainment programmes to see to, holly and so forth to get ordered, statements of outstanding accounts to write for, tips to pay, arrangements of all kinds to make—in sum, a good deal of extra work and bother. And at public sanatoriums we enjoy in return no heart-warming spectacles such as that just mentioned. One's adult charges are not so pleasing to view. Sunday clothes and hair oil and cheap cigars, together with the flushed expansiveness induced by an unaccustomed ration of alcohol, adulterate the charm of the happiness on their more sophisticated faces. All the same, one must cultivate one's garden—set to, that is, and give the inmates what they have been used to every previous December and January of their lives.

On the 24th, then, we begin with a dinner to the men employed on the place: to the caretaker, to the engineer with his fireman, to the joiner, to the handy-man, and to the lad who works in the garden—six in all. Who is to take the head of the table? Not oneself; we will not, in Butler's shrewd words, so overweight our powers of conversation, and act as a wet blanket. Not the matron; feminine charm is here out of place. Neither of the two first-named functionaries either; their positions are so nearly equal as to make it unwise to plump for the seniority of one or other. But these two Kings of Brentford will both good-humouredly yield to a temporary regent in the person of the handy-man, who, by virtue of being a natural comedian in a small way, occupies a privileged position. A box of cigars, a dozen of beer, and one bottle of whiskey, with a gramophone, give them all a happy evening. Like the Italian writing a sonnet on sonnet-writing, we have got through a third of the task at one rush.

Christmas Day dinner almost manages itself, thanks to the crackers and the grateful sense of liberty conferred by suspending all but the most necessary restrictions and routine; thanks again, too, to that invaluable labour-saving gramophone. The carols we had before breakfast; thanks for them to the whole female staff. Rather more trouble is entailed by the whist-drive on Boxing Night. The matron distributes the prizes; and hot soup and biscuits send all to bed warm and satisfied. So much of a success is it that the patients petition for another—to be paid for and arranged by themselves—at the New Year. Answer—" Granted, but you must have it in the afternoon, so as not to give the engineers another late night." It comes off, successfully, too, although one must be on the watch to allay any beginnings of disputes. Now there is only the finale to think of, in the shape of the maids' ball.

Our active and obliging joiner is the man for this. Six feet high, thirteen stone, and something of a high jumper, he naturally commands respect as a Master of the Ceremonies. He knows the local talent and secures a pianist and a fiddler, who discourse a popular programme of dances, among them interesting country jigs and preposterous " Circassian Circles." He leads off gallantly with the matron, followed by the nominal head of the institution embracing a terribly weighty cook. The postman and cobbler and so forth whirl round with the nurses (not at all on their dignity) and the various maids. One smokes a great number of cigarettes and keeps handy in the vicinity until 12 o'clock, the hour of release. Everything is done until another season comes round, and eighty or ninety people have been entertained for about a five-pound note, which one's committee gladly grants.

Now, is not this better than elaborate theatricals with their long waits, and Christmas trees, which latter often make a sad hole in the shallow pockets of ward sisters? But impious questions such as this must not be multiplied, for if I criticise the *Damenwelt* of our hospital nursing system the Editor will be down upon me. No more at present, then, than this little picture of Christmas at a working-class sanatorium.

